# Omics-based analyses revealed metabolic responses of *Clostridium acetobutylicum* to lignocellulose-derived inhibitors furfural, formic acid and phenol stress for butanol fermentation

**DOI:** 10.1186/s13068-019-1440-9

**Published:** 2019-04-27

**Authors:** Huanhuan Liu, Jing Zhang, Jian Yuan, Xiaolong Jiang, Lingyan Jiang, Guang Zhao, Di Huang, Bin Liu

**Affiliations:** 1 State Key Laboratory of Food Nutrition and Safety, (Tianjin University of Science & Technology), Tianjin, 300457 China; 2 Key Laboratory of Food Nutrition and Safety, (Tianjin University of Science & Technology), Ministry of Education, Tianjin, 300457 China; 3 TEDA School of Biological Sciences and Biotechnology, Nankai University, TEDA, Tianjin, 300457 China; 4 Qingdao Institute of Bioenergy and Bioprocess Technology, Chinese Academy of Sciences, Qingdao, 266101 China

**Keywords:** *Clostridium acetobutylicum*, Lignocellulose hydrolysate inhibitors, Biofuel, Proteomics, Metabolomics

## Abstract

**Background:**

*Clostridium acetobutylicum* is a model fermentative anaerobe for consolidated bioprocessing of lignocellulose hydrolysates into acetone–butanol–ethanol (ABE). However, the main inhibitors (acids, furans and phenols) ubiquitous in lignocellulose hydrolysates strictly limit the conversion efficiency. Thus, it is essential to understand the underlying mechanisms of lignocellulose hydrolysate inhibitors to identify key industrial bottlenecks that undermine efficient biofuel production. The recently developed omics strategy for intracellular metabolites and protein quantification now allow for the in-depth mapping of strain metabolism and thereby enable the resolution of the underlying mechanisms.

**Results:**

The toxicity of the main inhibitors in lignocellulose hydrolysates against *C. acetobutylicum* and ABE production was systematically investigated, and the changes in intracellular metabolism were analyzed by metabolomics and proteomics. The toxicity of the main lignocellulose hydrolysate inhibitors at the same dose was ranked as follows: formic acid > phenol > furfural. Metabolomic analysis based on weighted gene coexpression network analysis (WGCNA) revealed that the three inhibitors triggered the stringent response of *C. acetobutylicum*. Proteomic analysis based on peptide mass spectrometry (MS) supported the above results and provided more comprehensive conclusions. Under the stress of three inhibitors, the metabolites and key enzymes/proteins involved in glycolysis, reductive tricarboxylic acid (TCA) cycle, acetone–butanol synthesis and redox metabolism were lower than those in the control group. Moreover, proteins involved in gluconeogenesis, the oxidative TCA cycle, thiol peroxidase (TPX) for oxidative stress were significantly upregulated, indicating that inhibitor stress induced the stress response and metabolic regulation. In addition, the three inhibitors also showed stress specificity related to fatty acid synthesis, ATP synthesis, nucleic acid metabolism, nicotinic acid metabolism, cell wall synthesis, spore synthesis and flagellum synthesis and so on.

**Conclusions:**

Integrated omics platforms provide insight into the cellular responses of *C. acetobutylicum* to cytotoxic inhibitors released during the deconstruction of lignocellulose. This insight allows us to fully improve the strain to adapt to a challenging culture environment, which will prove critical to the industrial efficacy of *C. acetobutylicum*.

**Electronic supplementary material:**

The online version of this article (10.1186/s13068-019-1440-9) contains supplementary material, which is available to authorized users.

## Introduction

The traditional acetone–butanol–ethanol (ABE) fermentation based on *Clostridium acetobutylicum* is ranked as the second largest in industrial fermentation, behind bioethanol production. Biobutanol, a renewable energy fuel, has recently received considerable attention because of its higher energy density, higher combustion heat, better engine compatibility and less corrosivity than those of bioethanol [1]. As the environment deteriorates and food shortages expand, ABE fermentation using nongrain raw materials (sugar industry waste, lignocellulose, glycerin and syngas) as the substrates is being used by the biobutanol industry [2]. Among the potential substrates, lignocelluloses, such as corn stover, wheat straw, switchgrass and sweet sorghum slag, are the most attractive biomass raw materials due to their low price, wide range of sources and renewability [3]. The effective development of lignocellulosic materials will also significantly reduce the dependence on the petrochemical industry and improve the comprehensive utilization of agricultural waste.

However, due to the “recalcitrance” of lignocellulosic biomass [4], the raw materials are usually subjected to pretreatment under acidic, alkaline, steam and other harsh conditions and then hydrolyzed by cellulase into monosaccharides (mainly hexoses and pentoses) for microbial fermentation. It is believed that the pretreatment and hydrolysis of lignocellulosic feedstocks produce a range of substances that are toxic to microbial cells [5], such as furfural, 5-hydroxymethyl furfural, formic acid, acetic acid, phenol, catechol, ferulic acid, syringaldehyde, vanillin and coumarin [6, 7]. These inhibitors are generally classified into three categories according to their sources and properties, namely, furans, weak acids and phenols [5]. The presence of these compounds will inhibit cell growth, substrate utilization and product synthesis, thus greatly reducing the production efficiency of lignocellulosic butanol. There have been many reports on the pretreatment, hydrolysis, detoxification and fermentation of lignocellulose [6, 8, 9], but most of them focus on the optimization of hydrolysis and fermentation processes and adopt the hydrolysis methods, explosion conditions, and detoxification processes used in lignocellulosic ethanol production. Research on lignocellulose-derived inhibitors has focused on *Saccharomyces cerevisiae* and *Zymomonas mobilis* [10–12] and so on, which has promoted the tremendous development of the bioethanol industry.

To date, few natural strains can efficiently utilize lignocellulosic hydrolysate to produce butanol, and due to an insufficient understanding of the inhibition mechanism, butanol production is lower than that of traditional substrate fermentation by various simple sugars, starchy crops, etc. as the substrates [13]. In previous studies, furfural and 5-hydroxymethyl furfural promoted the growth of *Cupriavidus basilensis* [14] and *C. acetobutylicum* [15] at low concentrations; Furfural and 5-hydroxymethyl furfural (3 g/L) were found to be stimulatory rather than inhibitory to *C. beijerinckii* BA101, although the mixture of the two negatively affected the culture [16]; 1.0 g/L phenolic compounds inhibited up to 64–74% of *C. beijerinckii* cell growth and completely inhibited the butanol production [7]; Up to 80 mM sodium acetate promoted the growth of strain and stabilized butanol production by preventing the strain degeneration of *C. beijerinckii* [17]. In addition, *C. acetobutylicum* was more sensitive to formic acid compared with *C. beijerinckii* [18], and 0.1 M formic acid induced the “acid crash” and completely inhibited butanol production and cell growth in *C. acetobutylicum* [19]. Therefore, different strains have different tolerance levels and stress responses to inhibitors, and different raw materials or hydrolysis processes generate different amounts and species of inhibitors [6, 20], which creates problems in the elucidation of metabolic response to inhibiting compounds present in lignocellulose materials at different cellular levels (metabolites, proteins, etc.) to identify potential targets which could be addressed to alleviate inhibition. Therefore, a systematic investigation to ascertain the mechanism used by butanol-producing strain in the presence of lignocellulosic hydrolysate inhibitors is urgently needed.

The development of omics technology provides a theoretical basis for understanding the metabolic response mechanism of microorganisms under various conditions and eliminating the production bottleneck of target products. Great efforts have been made to dissect the metabolic mechanism of *C. acetobutylium* by transcriptomics [21, 22], proteomics [23, 24] and metabolomics [25, 26]. At the same time, the methods based on mathematical statistical analysis and bioinformation analysis have been developed and matured rapidly, such as principal component analysis (PCA) [27], partial least square regression (PLS regression) [28], weighted gene co-expression network analysis (WGCNA) [29, 30], gene ontology (GO) [31], KEGG pathway [32], all of which provide a strong technical foundation for data mining. Among them, WGCNA is a method for the construction of biological networks based on pairwise correlations between variables. It allows one to define modules (clusters), intramodular hubs, and network nodes with regard to module membership, to explore the relationships between co-expression modules, and to compare the network topology of different networks (differential network analysis) [29, 30]. The WGCNA method is widely used in the functional genomics, especially in the transcriptomic analysis for thousands of studies (data from www.scopus.com). Now, this method is gradually applied in metabolomic analysis such as metabolic alterations in multiple sclerosis [33], astaxanthin-producing *Haematococcus pluvialis* under various stress conditions [34], tacrolimus accumulation in *Streptomyces tsukubaensis* [35], identification of a metabolomic signature associated with feed efficiency in beef cattle [36], and so on.

In this study, we investigated the toxicity of major inhibitors (weak acids, furans and phenol) ubiquitous in lignocellulosic hydrolysate against cells and butanol production to determine the most stress-promoting inhibitors. Next, the intracellular metabolites with significant contributions related to fermentation phenotype were identified by metabolomic analysis. The stress and interference on intracellular metabolic modules induced by different inhibitors were analyzed by the WGCNA method. Subsequently, a proteomics strategy was implemented to analyze the changes in metabolic pathways and relevant functional modules, and the mechanism underlying the stress response of the strain was proposed. This work will provide rational guidance for the optimization of lignocellulose hydrolysis, strain evolution and fermentation process.

## Materials and methods

### Strain and culture medium

*Clostridium acetobutylicum* ATCC 824 was purchased from the American Type Center Culture Collection (ATCC, Maryland, USA).

Reinforced Clostridium medium (RCM) [37] was used for seed cultivation. Fermentation medium was P2 medium containing 50 g/L glucose, 0.5 g/L K_2_HPO_4_, 2.2 g/L ammonium acetate, 0.5 g/L KH_2_PO_4_, 0.2 g/L MgSO_4_·7H_2_O, 0.01 g/L FeSO_4_·7H_2_O, 0.01 g/L NaCl, 0.01 g/L MnSO_4_·H_2_O, 1 mg/L *p*-aminobenzoic acid, 1 mg/L vitamin B1, and 0.001 mg/L biotin.

### Cultivation of *C. acetobutylicum*

*Clostridium acetobutylicum* spores were heat shocked at 85 °C for 10 min and then quickly cooled in a water bath for 5 min to the room temperature. Seed cultivation was carried out in 250 mL Erlenmeyer flasks with a 200 mL working volume and a 1% (v/v) inoculation volume in the YQX-II anaerobic incubator (Shanghai Longyue Instrument Equipment Co., Ltd., China) for 24 h at 37 °C, and the OD_600_ of the seed broth was approximately 3.0, which could be used for fermentation inoculation.

Anaerobic ABE fermentation was carried out in a 3.5 L fermenter (NBS BIOFLO-2000, New Brunswick Scientific Co., Inc., USA) with an inoculation volume of 10% (v/v) and a working volume of 2.0 L. Before inoculation, the fermentation broth was flushed with sterile nitrogen (99.99%) to remove oxygen (0.5–1.0 L/min, 15–20 min). The stirring speed was maintained at 100 rpm (revolutions per minute), and the temperature at 37 °C. The cultivation pH was monitored by a pH electrode and maintained at 5.8 by 6 M ammonia and 6 M HCl.

### Determination of biomass and glucose

Biomass was determined by measuring the optical density (OD) of fermentation broth at 600 nm by a 752 spectrophotometer (Shanghai Precision & Scientific Instrument Co., Ltd, China) exactly according to the instrument instruction. The concentration of sugar (glucose only) was determined by a biosensor SBA 40C followed by the protocol supplied by the manufacturer (Biology Institute of Shandong Academy of Sciences, China).

### Determination of fermentation broth products

The concentrations of ethanol, acetone, butanol, acetic acid and butyric acid in the fermentation broth were determined by gas chromatography (GC) with isobutyl alcohol (purity > 98.0%) as the internal standard. Briefly, 1 mL fermentation broth was centrifuged at 8855×*g* for 5 min, and the supernatant was filtered through a 0.22 μm nylon filter (13 mm, Tianjin Keyilong Lab Equipment Co., Ltd), followed by GC analysis (430-GC, Bruker Daltonics Inc., USA) with a BW-SWAX capillary column (30 m × 0.32 mm ID × 0.25 m, Bruker Daltonics Inc., USA) and an FID detector. The oven temperature was programmed to be maintained at 80 °C for 2 min, then increased at a rate of 10 °C/min to 100 °C, further at a rate of 50 °C/min to 230 °C, and maintained for 2 min. The inlet and detector temperatures were 250 °C. The split ratio was 1:20. The gas ratio was carrier gas (nitrogen): supplemental gas (nitrogen): air: hydrogen = 1 mL/min: 20 mL/min: 30 mL/min: 35 mL/min.

### Tolerance test

The effects of exogenous inhibitors (furfural, 5-hydroxymethyl furfural, furfural, formic acid, acetic acid, phenol, furfuryl alcohol and furoic acid) on several important metabolic product yields (ethanol, acetone, butanol, acetic acid, butyric acid, sampling at the end of fermentation, 60 h) were investigated. Inhibitor solutions in a concentration range of 0.5 g/L, 1.0 g/L, 2.0 g/L, 3.0 g/L and 4.0 g/L were prepared separately and added at 12 h after the start of fermentation when it was in the initial logarithmic phase and there were enough bacteria for sampling (> 5.0 g wet weight/L fermentation broth). All the regents used for tolerance test were purchased from Sigma-Aldrich Co. Ltd. with chromatographic purity (> 98.0%).

### Sample preparation for metabolomic analysis by gas chromatography–mass spectrometry (GC–MS)

Sample quenching and extraction of intracellular metabolites for GC–MS analysis were carried out in accordance with the method described previously [38]. Ten milliliters of fermentation broth was quenched with fourfold volumes of − 40 °C 60% prechilled methanol, quickly mixed and stored in the prepared ice box to terminate the intercellular metabolic activities. All the above operations should be completed in approximately 5 min. Subsequently, the mixture was centrifuged (Eppendorf 5417R, Germany) at 6149×*g* for 5 min at − 20 °C and washed three times with 10 mL 4 °C phosphate-buffered solution (PBS, 137 mM NaCl, 2.7 mM KCl, 10 mM Na_2_HPO_4_, and 2 mM KH_2_PO_4_, pH = 7.4). The resulting cell pellet was resuspended in a 1.5 mL Eppendorf tube at − 40 °C in 50% (v/v) methanol solution and then thawed in an ice bath for 5 min. The mixture was blended with a vortex mixer for 20 s, and put into the liquid nitrogen to be re-freezed for 5 min and thawed for 1 min at − 20 °C. Three times of freezing–thawing operation was carried out. After being centrifuged at 6149×*g* (− 20 °C) for 5 min, a 200 μL aliquot of supernatant was mixed with 50 μL 0.2 mg/mL succinic acid-2,2,3,3-d4 (CAS No. 14493-42-6, Sigma-Aldrich Co. Ltd.) as the internal standard and then freeze dried at − 45 °C for 12 h (Alpha 1-2LD PLUS, Christ, Germany). Subsequently, the samples were derivatized with a two-step method described by Xia et al. [39] with slight modification. In detail, 50 µL of 20 mg/mL methoxylamine hydrochloride–pyridine solution was added into the sample, and then kept at 40 °C for 90 min. Subsequently, 80 µL *N*-methyl-*N*-(trimethylsilyl) trifluoroacetamide was added and mixed, and kept at 40 °C for 30 min. Data acquisition by GC–MS was performed by the system including A7683B automatic sampler, GC analyzer 6890N and 5975C MSD mass spectrometer (Agilent Technologies, Inc., USA) equipped with Agilent HP-5MS capillary column. The parameter settings of the GC–MS system were exactly consistent with those of Wang et al. [38].

### Analysis of mass spectrometry data

The online software MZmine-2.1 (http://MZmine.sourceforge.net/) [40] was used to process the data in batches, including deconvolution, noise reduction, retention time alignment, peak area integral determination and compound identification with an NIST 2010 mass spectrum database (http://webbook.nist.gov/chemistry/). All identified compounds were matched in the KEGG database (http://www.genome.jp/kegg/kegg2.html). Finally, the identified metabolite peak areas were standardized by dry cell weight and peak area of internal standard (succinic acid-2,2,3,3-d4), obtaining the relative intracellular abundance of each metabolite.

### Multivariate statistical analysis of metabolomics data

Partial least squares-discriminant analysis (PLS-DA) was carried out using the software platform SIMCA-p 11.5 (Umetrics AB, Sweden) for multivariate statistical analysis. The data import and multivariate statistical analysis of PLS-DA were completed followed by the Simca-p tutorial (https://landing.umetrics.com/downloads-simca).

### WGCNA based on metabolomic data

WGCNA is a systematic biological method for describing the patterns of metabolite association among different samples. It can be used to identify highly synergistic metabolite sets and to identify candidate biomarker genes or metabolic targets according to the connectivity of metabolite sets [34, 41]. WGCNA was built from the GC–MS datasets by calculating weighted Pearson correlation matrices relative to the relative metabolite abundance followed by the proposal provided by Pei and Wang et al. [34, 35, 42]. Briefly, connection strength matrices were first created from the transformation of weighted correlation matrices by a power function, and then used to calculate topological overlap (TO). The metabolites with highly similar correlation relationships were grouped into the same modules through hierarchical clustering based on the results of TO. Subsequently, average linkage hierarchical clustering was performed to obtain metabolite dendrograms. The module assignment determined by the Dynamic Tree Cut of WGCNA was distinguished with the color row underneath the dendrograms. Modules with correlation *r* ≥ 0.8 and statistical significance *P* value < 0.05 were extracted for further investigation.

### Preparation of the protein extracts

Intracellular protein extraction for two-dimensional electrophoresis (2-DE) was implemented followed by the previous work [43] with some modifications. Briefly, 10 mL fermentation broth was centrifuged at 4 °C, 8855×*g* for 10 min. The cell pellet was resuspended by 1 mL precooled PBS buffer (NaCl 137 mM, KCl 2.7 mM, Na_2_HPO_4_ 10 mM, and KH_2_PO_4_ 2 mM, pH 7.4, 4 °C), then centrifuged at 4 °C, 8855×*g* for 10 min, repeated five times. The cell pellet was ground in liquid nitrogen for cell disruption and then redissolved in 1 mL PBS buffer containing 1 mM phenylmethanesulfonyl fluoride (PMSF) and 0.1% DL-dithiothreitol (DTT). Cells were further broken by intermittent ultrasonication at the power of 200 W, 5 cycles of a 1-second pulse with a 2-second gap on ice using a UP200S cell sonicator (Hielscher Inc., Germany). Subsequently, cell debris was centrifuged (8855×*g*, at 4 °C for 30 min). A fourfold volume of 10% precooled trichloroacetic acid–acetone solution was added into the supernatant and maintained at − 20 °C overnight for protein precipitation. The protein pellet was then washed twice with prechilled 90% acetone aqueous solution and centrifuged at 8855×*g* for 30 min at 4 °C. After the protein pellet had been air dried at room temperature for 2–3 h, proteins were dissolved in lysis buffer (8 M urea, 2 M thiourea, 2% (w/v) 3-[(3-cholamidopropyl)dimethylammonio]-1-propanesulfonate (CHAPS), 2% (w/v) ampholyte, 1% DTT and 1 mM PMSF) and subjected to sonication as stated above. The protein concentration was measured by the Bradford method using bovine serum albumin (BSA) as the standard [44].

### Two-dimensional electrophoresis (2-DE)

Isoelectric focusing (IEF) was carried out using immobilized pH gradient (IPG) gel strips (17 cm, pH 4-7, linear, Bio-Rad) that were rehydrated in the rehydration solution (8 M urea, 2 M thiourea, 0.5% CHAPS, 0.52% ampholyte, 1% DTT and 0.02% bromophenol blue) for 12 h.

For the first dimension, extracts containing 800 µg protein were mixed with the same volume of rehydration solution and electrophoresed by the Multiphor II system (Amersham Biosciences, USA) at 20 °C for a total of 93,000 Vh (200 V for 4 h, 500 V for 1 h, 1000 V for 1 h, 1000–8000 V for 3 h, 8000 V for 76,000 Vh). For the second-dimension gel electrophoresis, IEF strips were first equilibrated for 15 min with equilibration buffer-I (50 mM Tris–HCl pH 6.8, 6 M urea, 30% glycerin, 2% SDS, 2% DTT and 0.02% bromophenol blue) and another 15 min with equilibration buffer-II (50 mM Tris–HCl pH 6.8, 6 M urea, 30% glycerin, 2% SDS, 2.5% iodoacetamide, 0.02% bromophenol blue). Then, the IPG strips were transferred onto 12% SDS-PAGE using Protean II Xi cells (Bio-Rad, USA). Gels were initially run at 15 mA for 15 min and then at 250 V per gel until the bromophenol blue migrated to the bottom of the gel. Subsequently, the gels were stained overnight with Coomassie brilliant blue staining solution (10% ammonium sulfate, 10% phosphoric acid, 0.12% G250 and 20% methanol). Then, the stained gels were heated in the boiling destaining solution (3% acetic acid-ddH_2_O solution) for 30 min and subjected to image analysis.

### Image analysis

Gels of SDS-PAGE were scanned at 600 dpi using a PowerLook 2100XL (UMAX Technologies Inc., USA) and imported into PDQuest software (version 8.01, Bio-Rad, USA) for analysis [43]. A *t* test with Benjamin–Hochberg false discovery rate (FDR) correction for multiple testing was performed for the statistical analysis of the changes in protein abundance. The protein spots meeting the following three criteria were treated as differentially expressed protein (DEP) spots: I) average abundance changes (treated group vs. control group) were greater than 1.5-fold or less than 0.67-fold, II) protein spots were detected in all three parallel samples, and III) the fold change with an adjusted *P* value < 0.05 by a *t* test with FDR correction was considered to be significant. The DEPs were subjected to identification by matrix-assisted laser desorption ionization-time of flight/time of flight-mass spectrometry (MALDI-TOF/TOF-MS).

### MS analysis and database search

Protein spots in SDS-PAGE were carefully excised from Coomassie brilliant blue-stained gels and subjected to in-gel trypsin (from porcine pancreas, proteomics grade, Sigma-Aldrich Co. Ltd.) digestion according to Shevchenko’s method [45]. An ABI 4700 Proteome Analyzer (Applied Biosystems, USA) with the positive ion acquisition mode was used, and the collection range was 700–4000 Da. The laser intensity was 1000. GPS explorer 3.0 (Global Proteome Server, Applied Biosystems, USA) was used for MS data analysis. The protein database of *C. acetobutylicum* ATCC 824 was downloaded from NCBI (National Center for Biotechnology Information) (https://www.ncbi.nlm.nih.gov/protein?term=txid1488[Organism]) as the reference. The database search was implemented as Wang et al. described [46], and a protein with confidence coefficient Pi ≥ 95% was acceptable.

To reduce the analytical error, each metabolome experiment was biologically repeated four times, and proteome experiments were biologically repeated three times.

## Results and discussion

### Fermentation characteristics under the stress of lignocellulose-derived inhibitors

**Fig. 1:**
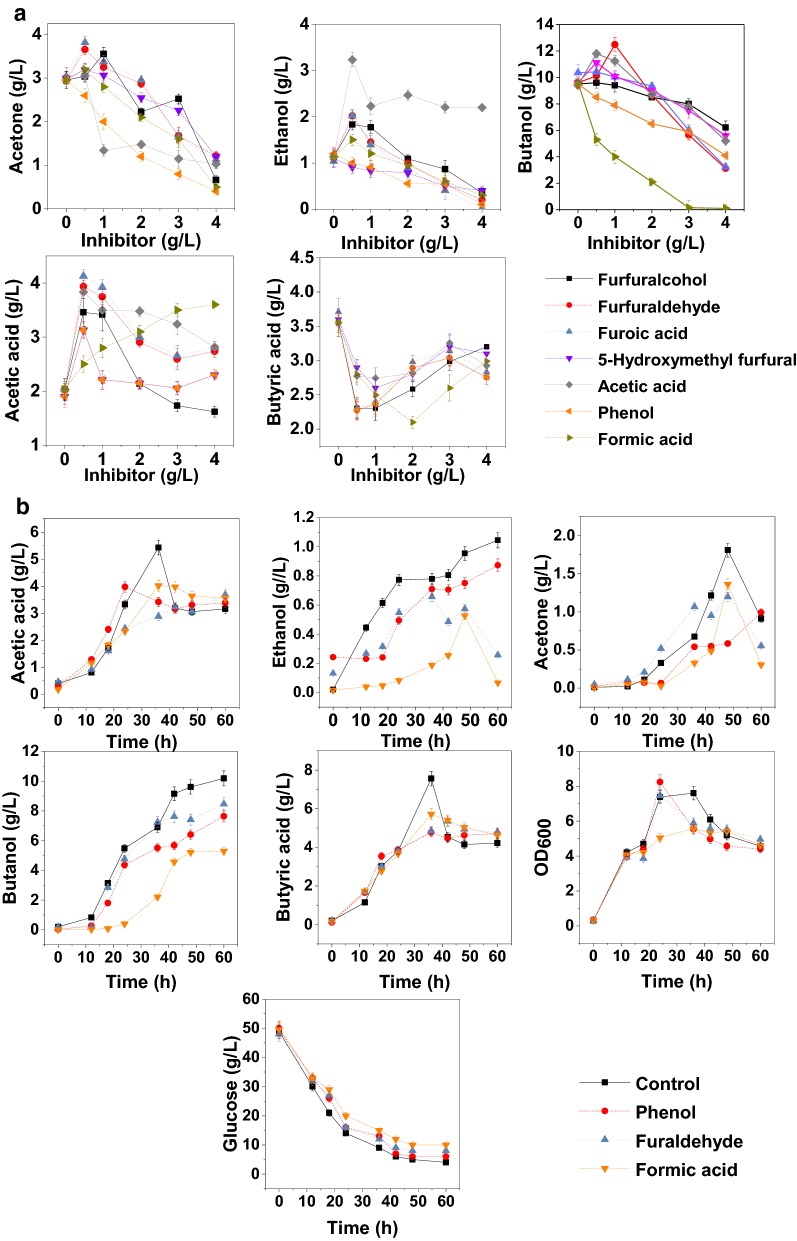
Effects of furans, organic acids and phenol on the fermentation performance of *C. acetobutylicum*. Tolerance test (**a**) and time course fermentation characteristics (**b**) under the stress of typical lignocellulose-derived inhibitors. Errors bars stand for the standard deviations calculated from three experimental replicates

To investigate the fermentation performance of *C. acetobutylicum* in the presence of inhibitors, furfural, 5-hydroxymethyl furfural, furfural, formic acid, acetic acid and phenol were selected as the typical inhibitors for the tolerance test. In this study, butanol yield was used for the main evaluation index of the inhibition effect on fermentation performance. As shown in Fig. 1a, with the increase of inhibitor concentration, the final concentration of butyric acid in the treated group firstly decreased and then increased. At higher concentrations (2.0–4.0 g/L for formic acid, while 1.0–3.0 g/L for other conditions), the butyric acid production increased, probably because more ATP was consumed to resist the adverse external environment [21, 47] rather than to absorb the butyric acid to produce butanoyl phosphate. For acetic acid production, as shown in Fig. 1a, with the increase of inhibitor concentration, the final concentration of acetic acid in the treated group firstly increased and then decreased, which was just the opposite of the butyric acid panel.

It was worth noting that the addition of phenol and formic acid at a low concentration (0.5 g/L) could greatly inhibit the production of butanol. In contrast, low concentrations of furfural, 5-hydroxymethyl furfural, acetic acid, furfuryl alcohol and furoic acid could improve fermentation performance. At high concentrations of inhibitors, *C. acetobutylicum* could not maintain normal metabolism, and ABE production was hindered. As shown in Fig. 1a, the inhibition of furfuryl alcohol and furoic acid at high concentrations (> 2 g/L) was less than that of furfural, probably due to the detoxification mechanism of furfural, though which furfural can be reduced to furfuryl alcohol [48] or converted into furoic acid through the furfural oxidation pathway [49]. Thus, both furfuryl alcohol and furoic acid were less harmful to *C. acetobutylicum* cells than furfural.

Both acetic acid and formic acid can be produced by *C. acetobutylicum* [50], but the effect of acetic acid and formic acid on *C. acetobutylicum* had a big difference. In this study, the butanol production was maintained when the medium was added by up to 2.0 g/L acetic acid. However, the addition of 0.5 g/L formic acid significantly lowed the butanol production (Fig. 1a). It was reported that the addition of only 1 mM formic acid to corn mash medium led to an “acid crash”, a phenomenon which occasionally occurs in pH-uncontrolled batch fermentations resulting in premature cessation of ABE production [51], while fermentation continued even when acetic acid and butyric acid concentrations increased up to 90 mM [19], indicating that ABE fermentation is highly sensitive to formic acid, which is consistent with the present results.

Above all, as shown in Fig. 1a, the toxicity of lignocellulose hydrolysate inhibitors at the same dose was ranked as follows: formic acid > phenol > furfural. Other inhibitors exhibited somewhat inhibitory or promotional effects related to the type and dose of inhibitors. Therefore, furfural, formic acid and phenol were selected as the main inhibitors to investigate the dynamic changes in the fermentation characteristics under the stress of lignocellulose hydrolysate inhibitors and the concentrations of these inhibitors were set at sublethal dose: 1.5 g/L (Fur group) furfural, 0.5 g/L (For group) formic acid, and 0.5 g/L (Phe group) phenol.

Figure 1b shows the fermentation characteristics under the stress of these three inhibitors. At 15 h (3 h after the addition of inhibitors), biomass growth (OD_600_) slowed down significantly, but recovered at 18 h (6 h after the addition of inhibitors), and the biomass of the Phe group (0.5 g/L) was even greater than that of the control group at 24 h. After 24 h, the biomass synthesis, glucose consumption rate as well as the production and reutilization of acetic acid and butyric acid in control group were higher than those of the treated groups (Fig. 1b). Therefore, for butanol production, 0.5 g/L formic acid showed the strongest inhibitory effect, followed by 0.5 g/L phenol and then 1.5 g/L furfural.

Subsequently, we adopted an integrated proteome and metabolome strategy to systematically elucidate the stress response caused by the addition of exogenous inhibitors to *C. acetobutylicum*. In this study, 1.5 g/L furfural, 0.5 g/L formic acid and 0.5 g/L phenol were added at 12 h for omics experiments. Proteome sampling points were set at 18 h and 36 h of fermentation, while the metabolome sampling points were at 15 h, 18 h, 24 h and 36 h.

### Metabolome analysis

**Fig. 2:**
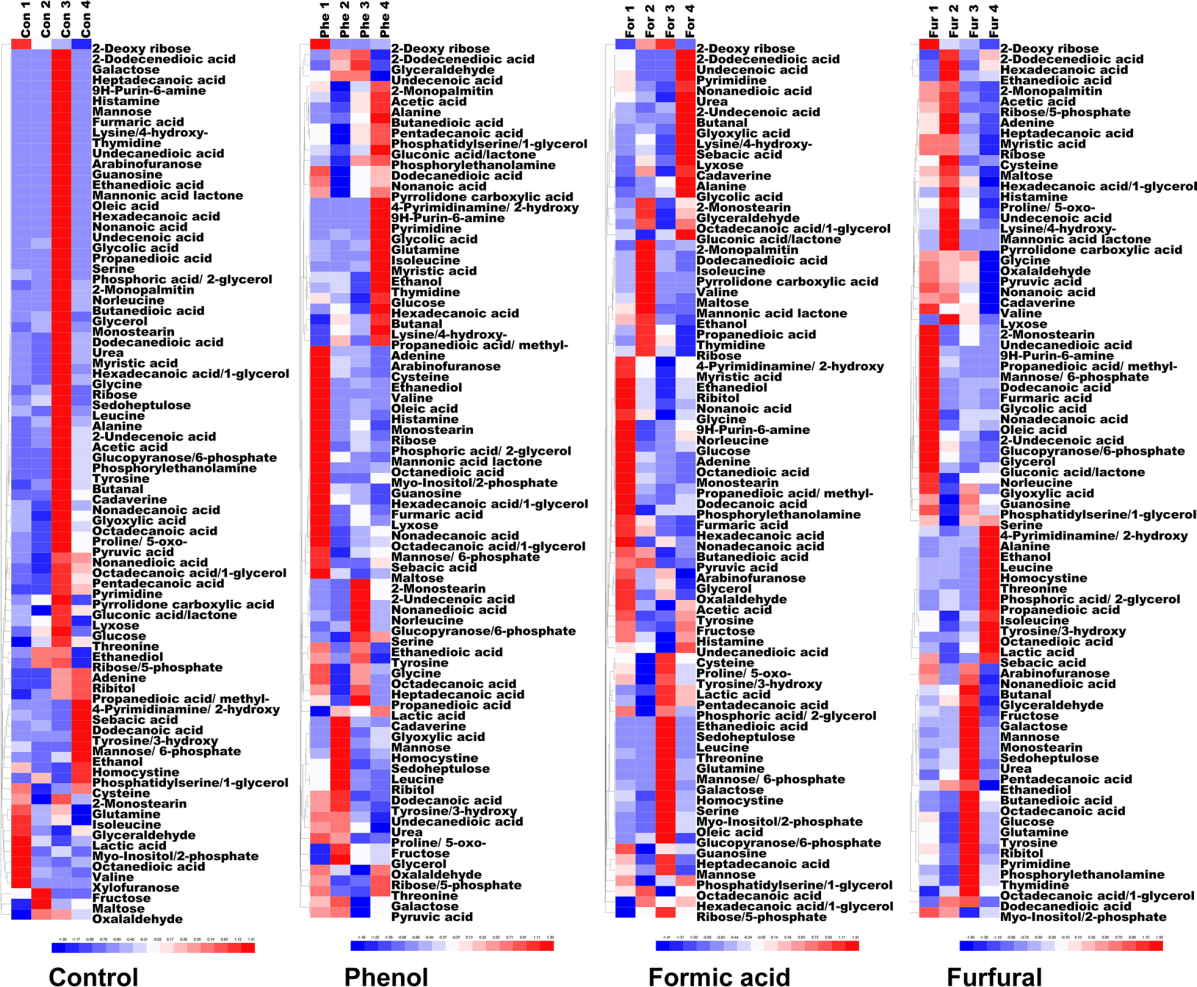
Heat map analysis of the intracellular metabolome. Relative abundances of intracellular metabolites are processed by zero-mean normalization. All the metabolites are ordered by hierarchical cluster analysis. Suffixes _1, _2, _3 and _4 stand for the sampling time of 15 h, 18 h, 24 h and 36 h

The intracellular metabolites of *C. acetobutylicum* were detected by GC–MS. A total of 84 intracellular metabolites were identified, including amino acids, fatty acids, sugars, alcohols and a small number of phosphate compounds (Additional file 1). Changes in the metabolite profiles at the sampling points are shown in Fig. 2. Obviously, in the control group, most of the metabolites had the largest relative abundances at the third time point (24 h), while in the inhibitor-treated groups, the changes of metabolite abundances with time course were highly correlated with the metabolic species. After 24 h, in the treatment group, due to the decrease of intracellular metabolite abundances, *C. acetobutylicum* cells lost the growth potential and entered the decline stage quickly; therefore, the biomass gradually decreased as shown in Fig. 1b. However, in the control group, the biomass maintained a long period from 24 to 36 h. The significant changes of intracellular metabolic profiles in inhibitor-treated groups indicated that the three inhibitors triggered the stringent response of *C. acetobutylicum*. At 36 h of fermentation, the intracellular metabolite abundance increased somewhat under the three conditions, indicating that the cells adapted to inhibitor stress by adjusting intracellular metabolism.

**Fig. 3:**
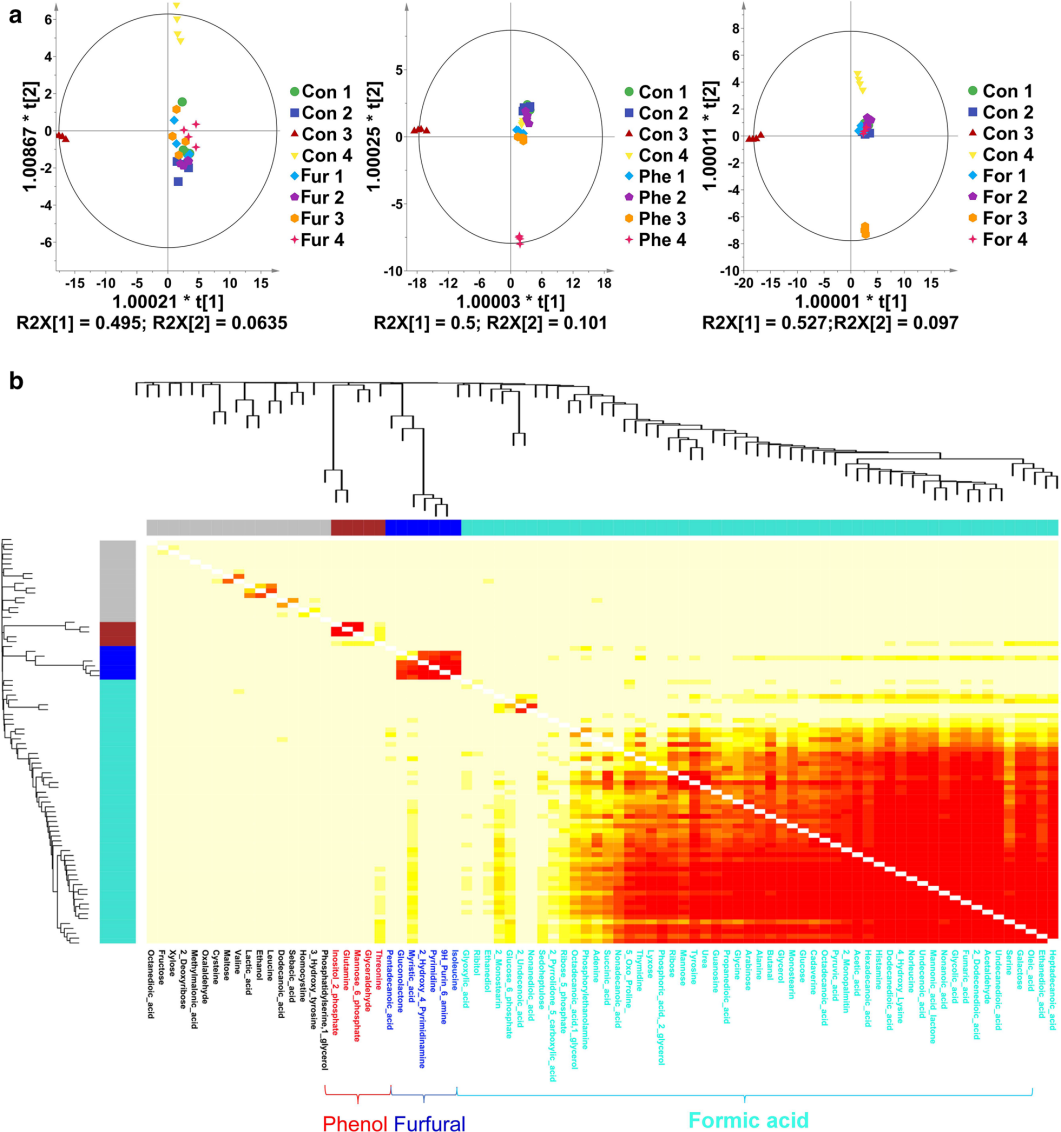
PLS-DA modeling (**a**) and WGCNA analysis (**b**) of the intracellular metabolome under the stress of furfural, phenol and formic acid, respectively. The cyan module corresponds to the formic acid condition, the blue module corresponds to furfural condition, and the red module corresponds to phenol condition

To explore the changes in intracellular metabolites under inhibitor stress, the obtained metabolomic data were subjected to PLS-DA analysis and the results were shown in Fig. 3a. The four parallel samples gathered in the score plot showed that the samples in each experimental group had high repeatability, and the distinct separation between the eight groups indicated that there were significant differences between the sample groups at each time point; these differences can be used for further data analysis [52].

To analyze intracellular metabolic responses, WGCNA method was adopted to process the GC–MS dataset to identify metabolic modules that were highly associated with inhibitor stress. As shown in Fig. 3, three metabolic modules (Red Module, Cyan Module and Blue Module) were identified, related to the Phe, For and Fur conditions, respectively.

The metabolite species in the metabolic modules under different inhibitors differed greatly. In the Phe group, the metabolic module mainly contained threonine, glyceraldehyde, mannose-6-phosphate, glutamine and inositol-2-phosphate, which are mainly involved in glucose metabolism and amino acid metabolism. Glutamic acid is a hub metabolite in amino acid metabolism related to the TCA cycle and is an important precursor of glutamine, proline, arginine and lysine; threonine can be converted to other metabolic intermediates, such as butyryl-CoA, succinyl-CoA, serine, and glycine. The other three metabolites are all related to carbohydrate metabolism, suggesting that glucose metabolism is influenced by the addition of phenol. In the Fur group, the metabolic module mainly included isoleucine, 9H-purin-6-amine, pyrimidine, 2-hydroxy-4-pyrimidinamine, myristic-acid, gluconolactone, and pentadecanoic acid, which are mainly involved in nucleic acid metabolism, fatty acid metabolism, leucine metabolism and the pentose phosphate pathway. It was worth noting that in the For group, up to 55 metabolites in the metabolic modules clustered by WGCNA were closely related to formic acid stress, covering more than half of the detected metabolic species. These metabolites are involved in a lot of intracellular metabolic pathways and were significantly different from the metabolic response to furfural and phenol stress. Figure 1b shows that the biomass, ABE production, and acetic acid and butyric acid production of the For group were lower than those of the control group from 24 to 36 h, indicating that formic acid triggered a much stronger stringent response than did the other two inhibitors. Thus, the intracellular metabolic adjustment and adaptation differed from those under the other two conditions.

### Proteome analysis

In this study, a total of 185 DEP spots were obtained. Protein information (Spot No., Protein Name, Accession Number, Peptide Count, Protein Score, Average, Standard Deviation, Fold Change, *P* value, FDR, adjust Fold Change) was supplied in Additional file 2. These DEPs covered a large portion of the intracellular metabolic processes of *C. acetobutylicum*, suggesting that metabolism changes significantly under the stress of these three inhibitors.

#### Central carbon metabolism

**Fig. 4:**
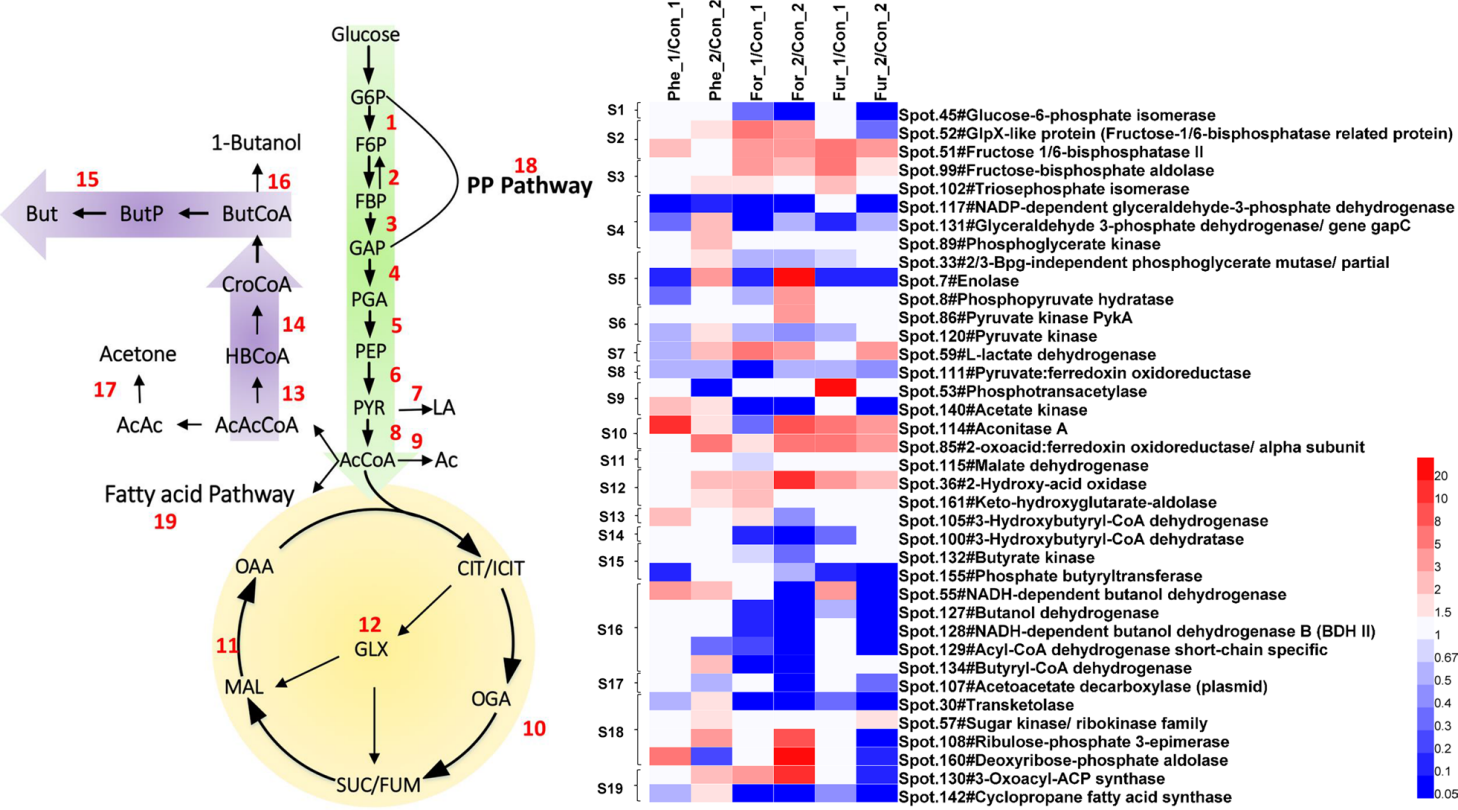
Changes in central carbon metabolism-related proteins under the stress of furfural, phenol and formic acid, respectively. Left is a schematic diagram of the central metabolism and right is a heat map of differentially expressed proteins. All the values are averages of the protein abundances in three samples. Suffixes _1 and _2 stand for the sampling time of 18 h and 36 h

Central carbon metabolism is the most important intracellular metabolic module and provides abundant carbon skeletons, energies (ATP, reducing equivalents) and ABE precursors. Here, a total of 19 metabolic steps and 38 protein spots involved in the Embden–Meyerhof–Parnas (EMP) pathway, pentose phosphate (PP) pathway, TCA cycle, fatty acid metabolism, butanol synthesis, and two-carbon metabolism (glyoxylate cycle) were gathered in Fig. 4.

In the EMP pathway (S1 to S9), glucose-6-phosphate isomerase (Spot 45) was downregulated in group For_1, 2 and Fur_2. It was worth noting that abundances of the two enzymes, 1,6-diphosphate fructose phosphohydrolase and GlpX-like proteins (Spots 51, 52 in Step 2) which were involved in the hydrolysis and regeneration of fructose phospholipid bonds of 1,6-diphosphate in the gluconeogenesis pathway [53, 54], together with fructose-diphosphate aldolase and triose phosphate isomerase in Step 3, were upregulated or had no significant changes under the stress of the three inhibitors than did the control group (Fig. 4), suggesting that gluconeogenesis metabolism was active and glucose catabolism was blocked. In a previous report, both the ClpX protein and fructose-diphosphate aldolase were upregulated under butanol stress, and the expression of fructose-diphosphate aldolase was positively correlated with the concentration of butanol [21], indicating that fructose-diphosphate aldolase played an important role in the stress response.

In Step 4, glyceraldehyde-3-phosphate can be converted into glycerate-3-phosphate under the catalysis of the three enzymes, NADP-dependent glyceraldehyde-3-phosphate dehydrogenase (Spot 117, GapN), glyceraldehyde-3-phosphate, hydrogenase (Spot 131, GapC) and glycerate phosphate kinase (Spot 120, Pgk). GapN can directly convert glyceraldehyde 3-phosphate to 3-phosphoglycerate, while the latter two enzymes achieve the same function in two steps: GapC converts glyceraldehyde 3-phosphate to 1,3-diphosphoglycerate and then produces 3-phosphoglycerate by Pgk accompanied by the generation of an ATP molecule, which is generally considered to be irreversible and the rate-limiting step in the EMP pathway. In Fig. 4, GapN and GapC (except in group Phe_2) in Step 4 were significantly downregulated under the three conditions, indicating the pathway inhibition from glyceraldehyde-3-phosphate to glycerate-3-phosphate. In addition, it could be found that Step 4 was subjected to more serious stress under the condition of formic acid.

S5 contains a 3-diphosphoglycerate mutase and two enolase enzymes (Spot 33, 7, 8), through which glycerate-3-phosphate is converted to phosphoenolpyruvate (PEP). The three enzymes were present at low abundances at 18 h and upregulated at 36 h in the Fur and Phe groups (Fig. 4).

Pyruvate kinase (Spot 86, 120) in S6 catalyzes the formation of pyruvate and ATP from PEP, which is the third rate-limiting step in the EMP pathway. The relative abundance of Spot 120 was lower than that of the control group at 18 h and recovered at 36 h (except in group For_2).

S7 contains l-lactate dehydrogenase (Spot 59), which converts intracellular pyruvate to L-lactic acid. The abundance of L-lactate dehydrogenase of treated groups was significantly higher than that in the control group (except in group Fur_1 and Phe_2). As shown in Additional file 1 of metabolome data, the intracellular lactic acid abundance in the late phase of fermentation (24 h and 36 h) was significantly higher than that in the control group, probably due to the upregulation of L-lactate dehydrogenase.

In S8, pyruvate: ferredoxin oxidoreductase (PFOR, CA_C2229, Spot 111), the enzyme responsible for the conversion of pyruvate to acetyl-CoA, plays an important role in the conversion of acidogenesis to solventogenesis by regulating redox metabolism. The active site of PFOR consists of an iron sulfur cluster that transfers electrons to ferredoxin by pyruvate dehydrogenation. PFOR of *C. acetobutylicum* is highly susceptible to oxygen stress [55]. As shown in Fig. 4, the abundance of PFOR was only approximately 0.5-fold that of the control group at 18 h (group Phe_1, For_1 and Fur_1) under the stress of three inhibitors, while at 36 h, the relative abundance of the enzyme was only 0.04-fold in group For_2 and was almost completely inhibited, suggesting that the pathway from pyruvate to acetyl-CoA was severely blocked.

S9 is involved in acetic acid anabolism and contains two proteins, phosphoacetyltransferase (PTA, Spot 53) and acetate kinase (AK, Spot 140). As shown in Fig. 4, these two proteins were upregulated or had no significant changes at 18 h in group Fur_1 and Phe_1, while in group For_1 and For_2 AK were obviously downregulated.

It is worth noting that in the For condition the proteins of S1, S2, S3, S8 and S9 had more significant changes than the other two conditions. This difference might explain why formic acid was more toxic than the other two inhibitors to the strain.

The TCA cycle of *C. acetobutylicum* is cleaved at the node of succinic acid and thus split into the oxidative TCA pathway (S10) and the reductive TCA pathway (S11) [56, 57]. The DEPs involved in S10 are cis-aconitase (Spot 114) and 2-keto-ferredoxin oxidoreductase (Spot 85). The DEP in S11 is malate dehydrogenase (Spot 115), which catalyzes the conversion between malic acid and oxaloacetate. As shown in Fig. 4, the proteins in S10 were significantly upregulated in nearly all the treated groups, while protein in S11 was downregulated in group For_1 and had no significant changes in any other groups. The strengthened oxidative TCA and the weakened reductive TCA pathways might be related to the inhibition of S4 and S8 which limited the production of redox equivalents.

S12 is related to glyoxylate and dicarboxylate metabolism. 2-hydroxy acid oxidase (Spot 36) and ketohydroxyglutaric acid aldolase (Spot 161) catalyze the formation of pyruvic acid and glyoxylic acid by 4-hydroxyketoglutarate. As shown in Fig. 4, the two proteins were upregulated or had no significant changes in all the treated groups.

S13 to S17 showed that the protein expression profile of butanol–acetone synthesis pathway. 3-Hydroxybutyryl-CoA dehydrogenase (Spot 105) and 3-hydroxybutyryl-CoA dehydratase (Spot 100) is involved in the synthesis of butyryl-CoA. Butyrate kinase (Spot 132) and phosphoryl butyryltransferase (Spot 155) are involved in the transfer of butyryl group and the production of butyric acid. NADH-dependent butanol dehydrogenase B (BDH II, Spot 128), acyl-CoA dehydrogenase short-chain specific (Spot 129), and butyryl-CoA dehydrogenase (Spot 134) catalyze the formation of butyraldehyde from butyryl-CoA. NADH-dependent butanol dehydrogenase (Spot 55) and butanol dehydrogenase (Spot 127) catalyze the reduction of butyraldehyde to butano. Acetoacetate decarboxylase (Spot 107) is related to the production of acetone from acetoacetate. Obviously, formic acid showed the strongest toxicity to butanol–acetone synthesis pathway, followed by furfural and phenol. Most proteins under formic acid condition (For_1 and _2) were downregulated while in group Fur_1, Phe_1 and Phe_2 some proteins were even upregulated.

S18 is related to the PP pathway. Transketolase (Spot 30, TK) is involved in the interconversion of saccharide compounds (including C3, C4, C5, C6, C7 and so on) [58]. Figure 4 shows that TK in the PP pathway was significantly downregulated under the three stress conditions only except in group Phe_2. Glycokinase (Spot 57, KdgK, for phosphorylation of 2-keto-3-deoxy-d-glucose) and 2-keto-3-deoxy-d-gluconate-6-phosphate lyase (Spot 161) are the key enzymes of the Entner–Doudoroff (ED) pathway [59]. Spot 57 and Spot 161 (ED pathway) were upregulated or had no significant changes in all the treated groups. The activation of ED pathway in this study meant that the intracellular glycolysis pathway was blocked as reported previously [60]. In addition, ribulose phosphate mutase (Spot 108) catalyzes the interconversion between ribose-1-phosphate and ribose-5-phosphate, which links the PP pathway to the nucleic acid pathway. Deoxyribose-phosphate aldolase (Spot 160) can catalyze the cleavage of deoxyribose-phosphate aldolase to glyceraldehyde 3-phosphate and glyoxylate, which is also a linker between the PP pathway and dicarboxylate metabolism. The abundance changes of Spot 108 and Spot 160 were closely related to the species of inhibitors.

In summary, the key steps in the PP pathway (interconversion between C3 and C7) might be suppressed, while other parts of the PP pathway were upregulated. Inhibition of the PP pathway led to an insufficient supply of carbon precursors, hindering downstream metabolism, such as the synthesis of aromatic amino acids (requiring erythrose-4-phosphate and sedoheptulose-7-phosphate). Furthermore, this also led to a decrease in the abundance of intracellular aromatic amino acids (tyrosine and 3-hydroxytyrosine) (Additional file 1).

S19 is involved in intracellular fatty acid metabolism. Spot 130 is a β-ketoacyl-ACP synthase catalyzing the first extension reaction in the de novo anabolism of fatty acids [61], and it also plays an important role in the synthesis of branched-chain fatty acids [62]. Spot 142 is a cyclopropyl fatty acid synthase that transfers the activated methyl group in S-adenosylmethionine to a phosphatidyl fatty acid chain containing an unsaturated double bond to form a cyclopropane group, which is involved in the modification of fatty acids and the transfer of a one-carbon unit [63]. Figure 4 shows that the intracellular abundance of Spot 130 increased in group Phe_2 and For_2 while in group Fur_2 it was significantly downregulated, indicating that fatty acid anabolism was most active under phenol and formic acid stress. Meanwhile, Spot 142 was downregulated in all the treated groups except in group Phe_2. The downregulation of Spot 142 means that the unsaturated double bonds of phosphatidyl fatty acids could be retained, which would promote cell membrane fluidity and permeability and increase the toxicity of the inhibitor.

#### Amino acid and aminoacyl-tRNA synthesis

**Fig. 5:**
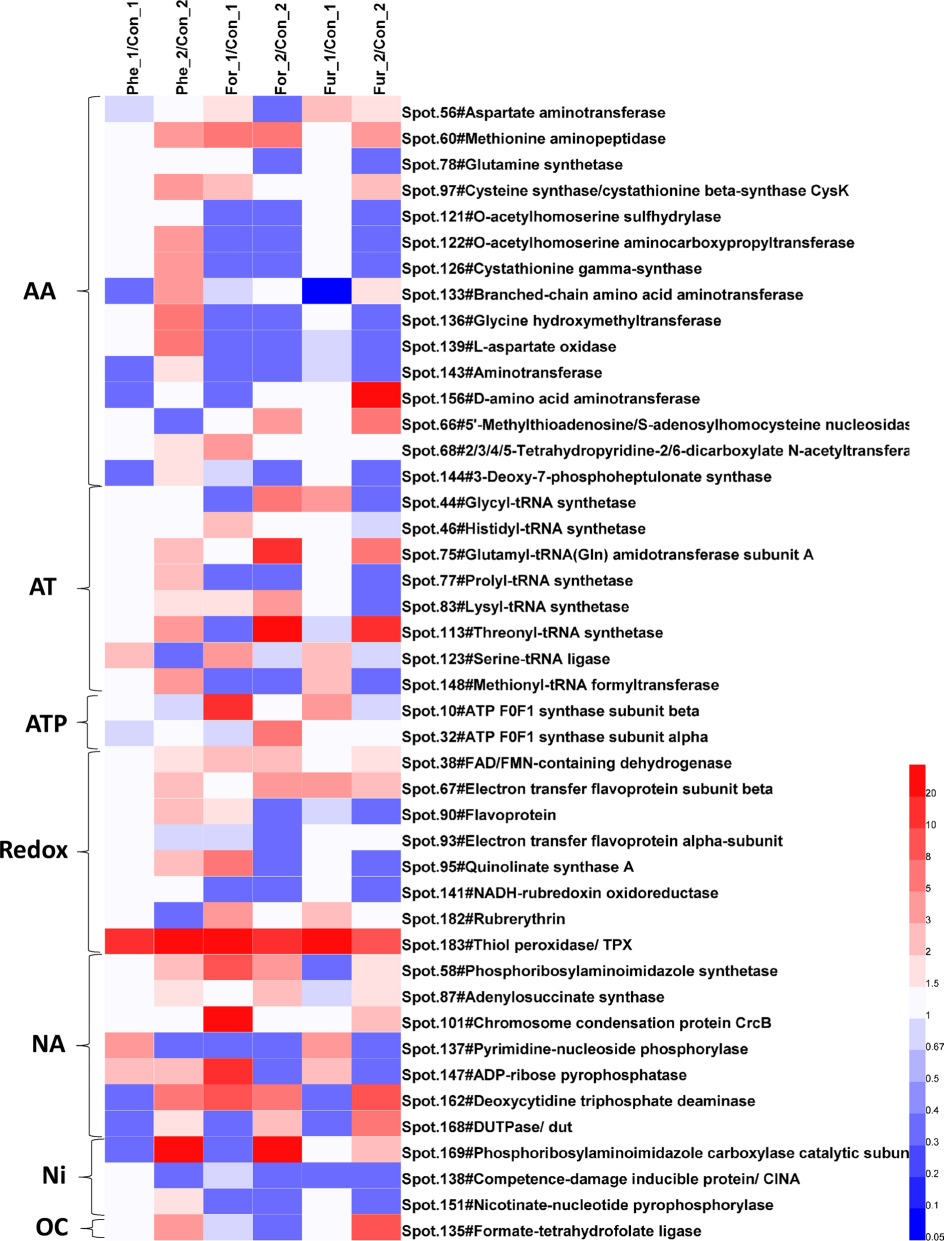
Abundance changes of the proteins involved in the amino acid and aminoacyl-tRNA biosynthetic pathways, nucleic acid, nicotinic acid, redox and ATP metabolisms. *AA* amino acid metabolism, *AT* aminoacyl-tRNA biosynthetic pathways, *ATP* ATP synthesis, *Redox* redox metabolism, *NA* nucleic acid metabolism, *Ni* nicotinic acid metabolism, *OC* one-carbon metabolism. All the values are averages of the protein abundances in three samples. Suffixes _1 and _2 stand for the sampling time of 18 h and 36 h

Amino acid and aminoacyl-tRNA synthesis are closely associated with the synthesis of intracellular proteins and polypeptides. As shown in Fig. 5, the expression pattern of enzymes related to amino acid synthesis in the Phe group was significantly different from that in the For and Fur groups. Only Spot 133 (branched amino acid aminotransferase), Spot 143 (aminotransferase), Spot 156 (d-amino acid aminotransferase) and Spot 144 (3-deoxy-d-arabino-heptulosonate 7-phosphate synthase, DAHP synthase) were downregulated in group Phe_1, while in group Phe_2 the expression of most proteins was higher than that in the control group. Among these proteins, DAHP synthase is the first key enzyme of the shikimate pathway and catalyzes the condensation of PEP with erythritol 4-phosphate to DAHP, thus controlling the carbon flux entry into the shikimic acid pathway [64]. DAHP synthase is usually inhibited by noncompetitive feedback of phosphate and amino acids such as tyrosine [65]. In Fig. 5, DAHP synthase was downregulated in group Phe_1 but upregulated in group Phe_2, while in the formic acid condition, DAHP synthase was downregulated in group For_1 and For_2. In the furfural condition, DAHP synthase had no significant change in group Fur_1 and was downregulated in group Fur_2. At the same time, the intracellular abundance of tyrosine (an aromatic amino acid) was at a low level (Additional file 1), which might be related to the inhibition from different conditions. Formic acid was the strongest inhibitor and, thus, the intracellular tyrosine level was the lowest in For groups.

Regarding aminoacyl-tRNA synthesis, Fig. 5 shows that intracellular aminoacyl-tRNA synthesis could be maintained only under the phenol stress and was significantly higher in group Phe_2 than that of the control. In the For group, the number of downregulated proteins decreased to 3 from 18 h to 36 h (For_1 to For_2), indicating that intracellular protein synthesis began to recover. In the Fur group, at 18 h (Fur_1), only Spot 113 (threonyl-tRNA synthetase) was significantly downregulated, but at 36 h most proteins were downregulated. Because aminoacyl-tRNA synthesis is involved in the intracellular synthesis of almost all proteins, up- or downregulation is strongly related to intracellular metabolic activities. In comparison, the synthesis of intracellular aminoacyl-tRNA under formic acid stress was significantly inhibited. Combined with analysis of the above central carbon metabolism, cells might slow down the metabolism under 0.5 g/L formic acid to survive and resist environmental stress. In addition, due to the upregulation of a number of proteins involved in amino acid and aminoacyl-tRNA synthesis under the phenol stress, cell growth could be maintained. Therefore, the biomass of phenol-treated group was higher than that of formic acid-treated group as shown in Fig. 1.

#### Energy and nucleic acid metabolism

In the present study, two ATP synthase subunits were detected, the α and β subunits (Spot 10, 32), both of which belong to the extracellular F1 unit of the F0F1 ATP synthase complex and contain nucleotide binding sites, wherein the binding site of the β subunit has the ATP synthesis or hydrolysis activities [66]. As shown in Fig. 5, the abundance changes of α and β subunits were different from each other under the three conditions, which were probably related to the maintenance of intracellular metabolic activities and transmembrane proton gradients [67, 68] since ATP synthase could pump out excess intracellular protons at the presence of ATP. The metabolic disturbance of ATP synthesis would also have an important impact on other ATP-consuming metabolic pathways, such as the synthesis of biomass, acetic acid, butyric acid and aromatic amino acid and so on.

In redox metabolism, it was worth noting that 3 proteins, Spot 38 (FAD/FMN-containing dehydrogenase), Spot 67 (electron transfer flavin beta subunit) and Spot 183 (thiol peroxidase, TPX), were upregulated under nearly all the treated conditions, especially TPX, whose expression was at least 8.7-fold higher than that in the control group. TPX plays an important role in responding to intracellular oxygen stress and scavenging oxygen free radicals [69]. As reported previously, erythropoietin (Spot 182) and TPX were significantly upregulated when *C. acetobutylicum* responded to extracellular oxygen stress [70], which was consistent with the results in this study. The above analysis suggested that the three inhibitors might all have effects similar to those of oxidative stress. Moreover, in Fig. 5 it could be found that the oxidation stress of formic acid and furfural was more significant.

Eight DEPs involved in nucleic acid and DNA metabolism, namely, Spot 58 (aminoimidazole nucleotide synthetase), Spot 87 (adenosyl succinate synthase), Spot 101 (chromosome lectin protein CrcB), Spot 137 (pyrimidine-nucleotide phosphatase), Spot 147 (ADP-ribose pyrophosphatase), Spot 162 (deoxycytidine triphosphate deaminase), Spot 168 (deoxyuracil-5-triphosphate nucleoside hydrolase) and Spot 169 (phosphoribosylaminoimidazole carboxylase) are given in Fig. 5. Among these proteins, Spots 58, 87, 147 and 169 are involved in the de novo synthetic pathway of purine, and Spot 87 (adenosyl succinate synthase) is the key enzyme catalyzing the conversion of IMP (inosine 5-phosphate) to AMP (nucleoside 5-phosphate) [71]. Spots 137, 162 and 168 are associated with pyrimidine synthesis. In addition, considering the one-carbon unit transfer in nucleic acid metabolism (e.g., converting UMP to dTMP) [72], Spot 135 (formate-tetrahydrofolate ligase, FTFL) is also discussed here within nucleic acid metabolism. Figure 5 shows that Spot 135 was downregulated in group For_1 and For_2, upregulated in group Phe_2 and Fur_2, suggesting that formic acid seriously inhibits one-carbon metabolism. At the same time, most proteins related to nucleic acid metabolism were upregulated in group Phe_2 suggesting that 0.5 g/L phenol was less stressful to nucleic acid metabolism than the other two inhibitors.

Nicotinic acid metabolism is the source of the intracellular NAD(P)H pool. Figure 5 shows that Spot 138 (nicotinamide-nucleotide amidase) was downregulated at 18 h and 36 h under the three conditions. Spot 151 (nicotinic acid-nucleoside pyrophosphatase, CINA) was upregulated in group Phe_2 but downregulated in group For_1, For_2 and Fur_2, indicating that the addition of formic acid also severely inhibited the synthesis of NAD(P)H, resulting in insufficient intracellular NAD(P)H. Although the upregulation of Spot 151 in group Phe_2 objectively alleviated the shortage of NAD(P)H caused by the decrease in CINA, the increase or decrease in these reductases generated a negative impact on the efficiency of ABE production since the cells were suffering the harsh environment under inhibitor stress.

#### Cell wall and membrane protein synthesis

**Fig. 6:**
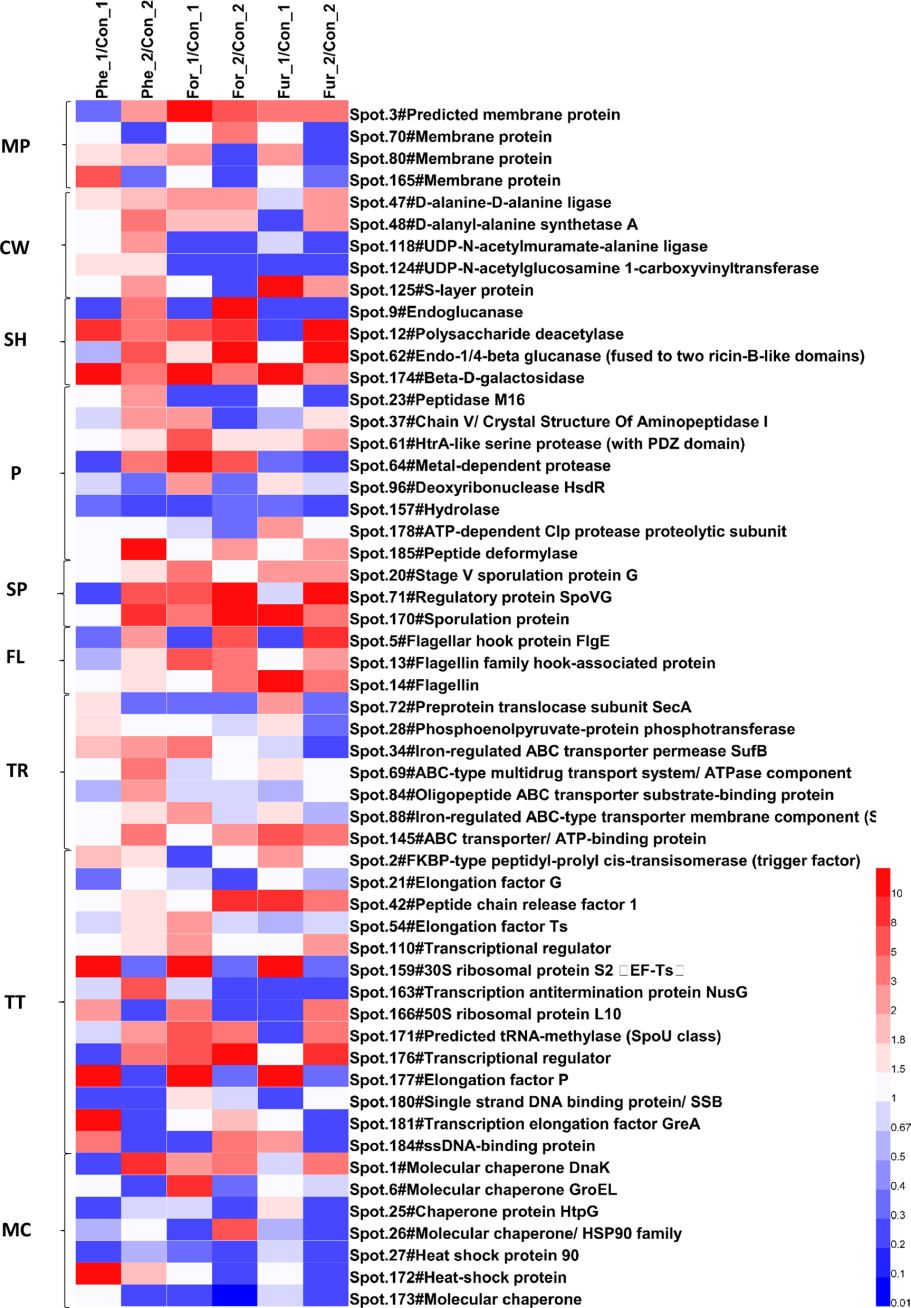
Abundance changes in the proteins involved in sporulation, flagella and ABC transporters and transcription, translation and molecular chaperones and so on. *MP* membrane protein, *CW* cell wall, *SH* hydrolase, *P* protease, *SP* sporulation related proteins, *FL* flagellar protein, *TR* transportation protein, *TT* proteins of transcription and translation, *MC* molecular chaperone. All the values are averages of the protein abundances in three samples. Suffixes _1 and _2 stand for the sampling time of 18 h and 36 h

Cell membranes and cell walls are important components that support cell morphology and protect cells from external environmental interference. As shown in Fig. 6, the abundances of membrane proteins and cell wall synthetic proteins under phenol stress were mostly higher than the control, indicating that cells adapted to phenol pressure by adjusting membrane proteins and changing cell wall components. Correspondingly, under formic acid and furfural stress, Spot 118 (UDP-*N*-acetylmuramate-l-alanine ligase) and Spot 124 (UDP-*N*-acetylglucosamine 1-carboxyvinyltransferase), the two enzymes catalyzing UDP-*N*-acetylglucosamine to peptidoglycan were downregulated in group For_1, For_2, Fur_1 and Fur_2, which could reduce the synthesis of peptidoglycan. In addition, the expression of two proteins, Spot 47 and Spot 48, for the elongation of peptidoglycan peptide chains recovered in the three conditions at 36 h, suggesting that the capacity of the cell wall synthesis was restored at the late period of fermentation.

Moreover, as shown in Fig. 6, most hydrolases, including glycoside hydrolases and protein/peptide hydrolases, were upregulated under the three conditions, except for Spot 9 (endoglucanase, the main component of cellulase) and Spot 23 (peptidase M16), suggesting that cells might maintain basic metabolism through degradation of intracellular macromolecules such as proteins, peptides and polysaccharides.

#### Spore formation, flagellum assembly and ABC transport system

As shown in Fig. 6, at 36 h the flagella and spore synthesis-related proteins were upregulated in all the treated groups. Considering that the synthesis of *C. acetobutylicum* spores was related to extracellular environment degradation (acid stress, solvent stress, etc.) [73–75], changes in spore synthesis-related proteins in this study might be related to environmental stress. The protein involved in flagellar assembly was significantly upregulated at 36 h, indicating that the cells restored the motor ability, thereby strengthening the intake of carbon and nitrogen sources.

As for the proteins associated with cell transport, the expression of SecA (Spot 72, preprotein translocator, in protein secretion) was inhibited in group Phe_2, For_1, For_2 and Fur_2, while in group Phe_1 and Fur_1 it was upregulated (Fig. 6). Most of the ABC transporters were upregulated under the phenol stress. The ABC transporter is on the plasma membrane containing two highly conserved ATP binding cassettes, which are dimerized by binding to ATP and then depolymerized by hydrolysis of ATP, and transfers the substrate to the other side of the membrane in association with the conformational changes. The upregulation of transporters indicates that the transfer of intracellular and extracellular metabolic fluxes was higher in the Phe group than that in the control group.

#### Transcription, translation and molecular chaperones

In this study, many transcription and translation regulatory proteins displayed significant changes in response to the stress of the inhibitors. Obviously, the expression of most proteins (8/14) increased in group For_1, followed by that in group Phe_1 (6/14) and Fur_1 (5/14) (Fig. 6). Spot 42 (peptide chain release factor 1, PrfA) is a bacterial release factor that recognizes the termination codon UAA and UAG to terminate protein synthesis. The high expression of PrfA might lead to the early termination of protein synthesis, resulting in protein synthesis disorders. Translation elongation factors could promote polypeptide chain extension in mRNA translation. Among the three translation elongation factors (Spot 21, Spot 54 and Spot 177), Spot 21 is an elongation factor G with transposase activity and Spot 54 is a heat-stable elongation factor Ts that promotes the formation of EF-Tu and EF-Ts dimers (EF-T) while Spot 177 (elongation factor P) activates the polypeptide transferase activity in the 70S ribosome and enhances the stability of peptide synthesis [76]. As shown in Fig. 6, elongation factor G was downregulated or had no significant changes in all the treated groups, while elongation factor Ts was upregulated in group Phe_2 and For_1. Interestingly, elongation factor P was significantly upregulated at 18 h but downregulated at 36 h under the three conditions, which was not directly related to the type of inhibitor.

Molecular chaperones can mediate the correct folding and assembly of other proteins. However, in this study, only heat shock proteins DnaK (Spot 1), HtpG (Spot 6) and GroEL (Spot 25) were significantly upregulated in several sample groups (Fig. 6) while other molecular chaperones were downregulated significantly, which promoted the toxicity of the inhibitor.

### Proposed metabolic mechanisms of *C. acetobutylicum* in response to lignocellulose-derived inhibitor stress for butanol fermentation

Above all, most of the intracellular metabolism pathways suffered from interference caused by the three inhibitors. WGCNA-based metabolomic analysis showed that the intracellular metabolite profiles changed significantly in response to external stress. Although there were different stress responses for each of the inhibitor conditions, an obvious decrease in metabolite abundance occurred at the same time (24 h), suggesting that generic and specific metabolic adjustment strategies were adopted by strains to cope with external stress.

**Fig. 7:**
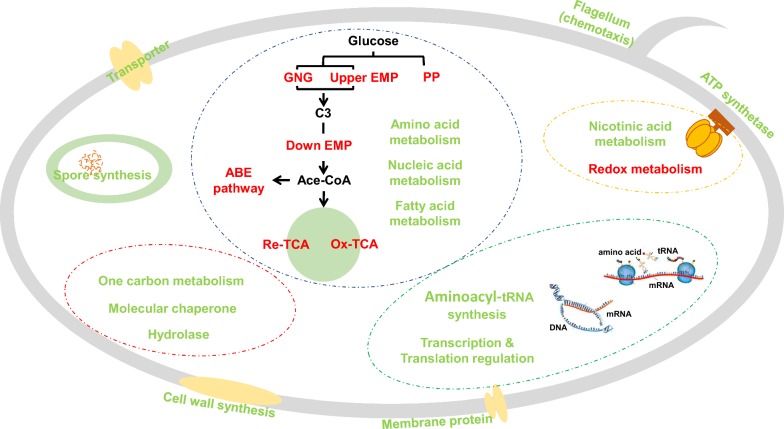
Metabolic response of *C. acetobutylicum* to lignocellulose-derived inhibitor stress for butanol fermentation. Font colors stand for the general (red) and the inhibitor-specific (green) responses to the inhibitor stress. *GNP* gluconeogenesis

As shown in Fig. 7, we drew a sketch of the intracellular metabolism and regulation when *C. acetobutylicum* was exposed to formic acid, furfural and phenol stress, and showed the general (in red font) and the inhibitor-specific (in green font) responses to the inhibitor stress. Under the stress of three inhibitors, the metabolites and key enzymes/proteins involved in glycolysis, reductive tricarboxylic acid (TCA) cycle, acetone–butanol synthesis and redox metabolism were lower than those in the control group. Moreover, proteins involved in gluconeogenesis, the oxidative TCA cycle, thiol peroxidase (TPX) for oxidative stress were significantly upregulated, indicating that inhibitor stress induced the stress response and metabolic regulation.

In previous study, 5-hydroxymethyl furfural and furfural are converted into furoic acid and 5-hydroxymethylfuroic acid through the oxidation pathway, in which 5-hydroxymethylfuroic acid is decarboxylated into furoic acid, and then into alpha-ketoglutarate, which finally enters the tricarboxylic acid (TCA) cycle in aerobic microbes [14]. In anaerobic microbes, 5-hydroxymethyl furfural and furfural are degraded into corresponding alcohols [15]. Due to the effect of furfural on redox metabolism and TCA cycle, the intracellular metabolic profile changed. As a result, the cells survived the inhibition of inhibitors, but the corresponding fermentation profile changed significantly.

Both acetic acid and formic acid can be produced by *C. acetobutylicum*. Pyruvate-formate lyase can convert pyruvate into acetyl-CoA and formic acid, and then formic acid is oxidized to carbon dioxide by formic acid dehydrogenase [50]. Formic acid showed the greatest inhibition to cells in this study. Most of the metabolic activities (from metabolomic and proteomic analysis) changed significantly in response to the formic acid stress, resulting in low fermentation performance.

As for phenol, generally, *C. acetobutylicum* does not produce or degrade phenols, but phenols can affect the proportion of proteins and lipids on the cell membrane, leading to the efflux of potassium ions and changing the function of the cell membrane [77]. Recently, the phenol inhibition mechanism of *Pseudomonas putida* KT2440 has been investigated; under phenol stress, the expression levels of proteins related to oxidative stress, general pressure response, energy metabolism and lipid metabolism were upregulated, indicating that the effect of phenols on intracellular metabolism is widespread [78], which is similar to the response of *C. acetobutylicum* to phenol stress in this study.

In addition, the three inhibitors also showed stress specificity related to fatty acid synthesis, ATP synthesis, nucleic acid metabolism, nicotinic acid metabolism, cell wall synthesis, spore synthesis and flagellum synthesis and so on.

## Conclusion

In summary, all three inhibitors showed similar inhibitory effects on central carbon metabolism but different effects on other metabolic and regulation modules. In this study, the toxicity of the main inhibitors in lignocellulose hydrolysates to *C. acetobutylicum* and ABE production was systematically investigated, and the changes in intracellular metabolism were systematically analyzed by metabolomics and proteomics. Integrated omics platforms have gained complementary systems and biological information that highlights the specific response of *C. acetobutylicum* to cytotoxic inhibitors released during the deconstruction and utilization of lignocellulose. Systematic analysis of the stress mechanism of these inhibitors will provide rational guidance for the optimization of lignocellulose hydrolysis, strain evolution and fermentation process.

## Additional files

**Additional file 1.** Metabolomic data.

**Additional file 2.** Proteomic data.

## Abbreviations

ABE: acetone–butanol–ethanol
MS: mass spectrometry
WGCNA: weighted gene coexpression network analysis
TCA: tricarboxylic acid
TPX: thiol peroxidase
PCA: principal component analysis
GO: gene ontology
RCM: reinforced Clostridium medium
OD: optical density
PBS: phosphate buffered solution
GC: gas chromatography
PLS-DA: partial least squares-discriminant analysis
TO: topological overlap
PMSF: phenylmethanesulfonyl fluoride
DTT: DL-dithiothreitol
CHAPS: 3-[(3-cholamidopropyl)dimethylammonio]-1-propanesulfonate
BSA: bovine serum albumin
2-DE: two-dimensional electrophoresis
IEF: isoelectric focusing
IPG: immobilized pH gradient
MALDI-TOF/TOF-MS: matrix-assisted laser desorption ionization-time of flight/time of flight-mass spectrometry
FDR: false discovery rate
GPS: global proteome server
DEP: differentially expressed proteins
PTA: phosphoacetyltransferase
AK: acetate kinase
GapN: NADP-dependent glyceraldehyde-3-phosphate dehydrogenase
GapC: glyceraldehyde-3-phosphate, hydrogenase
Pgk: glycerate phosphate kinase
PEP: phosphoenolpyruvate
PFOR: pyruvate: ferredoxin oxidoreductase
TK: transketolase
ED: Entner–Doudoroff
DAHP: 3-deoxy-d-arabino-heptulosonate 7-phosphate
IMP: inosine 5-phosphate
AMP: nucleoside 5-phosphate
FTFL: formate-tetrahydrofolate ligase
CINA: nicotinic acid-nucleoside pyrophosphatase
PrfA: peptide chain release factor 1
EF: elongation factor

## References

[1] Jin C, Yao MF, Liu HF, Lee CFF, Ji J (2011). Progress in the production and application of *n*-butanol as a biofuel. Renew Sustain Energy Rev.

[2] Ni Y, Sun Z (2009). Recent progress on industrial fermentative production of acetone-butanol-ethanol by *Clostridium acetobutylicum* in China. Appl Microbiol Biotechnol.

[3] Jang YS, Lee J, Malaviya A, do Seung Y, Cho JH, Lee SY (2012). Butanol production from renewable biomass: rediscovery of metabolic pathways and metabolic engineering. Biotechnol J.

[4] Herbaut M, Zoghlami A, Habrant A, Falourd X, Foucat L, Chabbert B, Paes G (2018). Multimodal analysis of pretreated biomass species highlights generic markers of lignocellulose recalcitrance. Biotechnol Biofuels.

[5] Palmqvist E, Hahn-Hagerdal B (2000). Fermentation of lignocellulosic hydrolysates. I: inhibition and detoxification. Bioresour Technol.

[6] Jonsson LJ, Carlos M (2015). Pretreatment of lignocellulose: formation of inhibitory by-products and strategies for minimizing their effects. Bioresour Technol.

[7] Cho DH, Lee YJ, Um Y, Sang BI, Kim YH (2009). Detoxification of model phenolic compounds in lignocellulosic hydrolysates with peroxidase for butanol production from *Clostridium beijerinckii*. Appl Microbiol Biotechnol.

[8] Jonsson LJ, Alriksson B, Nilvebrant NO (2013). Bioconversion of lignocellulose: inhibitors and detoxification. Biotechnol Biofuels.

[9] Chen H (2017). Technologies for biochemical conversion of biomass.

[10] Madhavan A, Srivastava A, Kondo A, Bisaria VS (2012). Bioconversion of lignocellulose-derived sugars to ethanol by engineered *Saccharomyces cerevisiae*. Crit Rev Biotechnol.

[11] Margeot A, Hahn-Hagerdal B, Edlund M, Slade R, Monot F (2009). New improvements for lignocellulosic ethanol. Curr Opin Biotechnol.

[12] Zaldivar J, Nielsen J, Olsson L (2001). Fuel ethanol production from lignocellulose: a challenge for metabolic engineering and process integration. Appl Microbiol Biotechnol.

[13] Qureshi N (2010). Agricultural residues and energy crops as potentially economical and novel substrates for microbial production of butanol (a biofuel). CAB Rev.

[14] Koopman F, Wierckx N, de Winde JH, Ruijssenaars HJ (2010). Identification and characterization of the furfural and 5-(hydroxymethyl)furfural degradation pathways of *Cupriavidus basilensis* HMF14. Proc Natl Acad Sci USA.

[15] Zhang Y, Han B, Ezeji TC (2012). Biotransformation of furfural and 5-hydroxymethyl furfural (HMF) by *Clostridium acetobutylicum* ATCC 824 during butanol fermentation. N Biotechnol.

[16] Ezeji T, Qureshi N, Blaschek HP (2007). Butanol production from agricultural residues: impact of degradation products on *Clostridium beijerinckii* growth and butanol fermentation. Biotechnol Bioeng.

[17] Chen CK, Blaschek HP (1999). Effect of acetate on molecular and physiological aspects of *Clostridium beijerinckii* NCIMB 8052 solvent production and strain degeneration. Appl Environ Microbiol.

[18] Cho DH, Shin SJ, Kim YH (2012). Effects of acetic and formic acid on ABE production by *Clostridium acetobutylicum* and *Clostridium beijerinckii*. Biotechnol Bioprocess Eng.

[19] Wang S, Zhang Y, Dong H, Mao S, Zhu Y, Wang R, Luan G, Li Y (2011). Formic acid triggers the “acid crash” of acetone–butanol–ethanol fermentation by *Clostridium acetobutylicum*. Appl Environ Microbiol.

[20] Palmqvist E, Hahn-Hägerdal B (2000). Fermentation of lignocellulosic hydrolysates. II: inhibitors and mechanisms of inhibition. Bioresour Technol.

[21] Tomas CA, Beamish J, Papoutsakis ET (2004). Transcriptional analysis of butanol stress and tolerance in *Clostridium acetobutylicum*. J Bacteriol.

[22] Janssen H, Grimmler C, Ehrenreich A, Bahl H, Fischer RJ (2012). A transcriptional study of acidogenic chemostat cells of *Clostridium acetobutylicum*-solvent stress caused by a transient n-butanol pulse. J Biotechnol.

[23] Mao S, Luo Y, Zhang T, Li J, Bao G, Zhu Y, Chen Z, Zhang Y, Li Y, Ma Y (2010). Proteome reference map and comparative proteomic analysis between a wild type *Clostridium acetobutylicum* DSM 1731 and its mutant with enhanced butanol tolerance and butanol yield. J Proteome Res.

[24] Sivagnanam K, Raghavan VG, Shah M, Hettich RL, Verberkmoes NC, Lefsrud MG (2011). Comparative shotgun proteomic analysis of *Clostridium acetobutylicum* from butanol fermentation using glucose and xylose. Proteome Sci.

[25] Zhao X, Condruz S, Chen J, Jolicoeur M (2016). A quantitative metabolomics study of high sodium response in *Clostridium acetobutylicum* ATCC 824 acetone-butanol-ethanol (ABE) fermentation. Sci Rep.

[26] Liu H, Huang D, Wen J (2016). Integrated intracellular metabolic profiling and pathway analysis approaches reveal complex metabolic regulation by *Clostridium acetobutylicum*. Microb Cell Fact.

[27] Kim S, Kang D, Huo Z, Park Y, Tseng GC (2018). Meta-analytic principal component analysis in integrative omics application. Bioinformatics.

[28] Palermo G, Piraino P, Zucht HD (2009). Performance of PLS regression coefficients in selecting variables for each response of a multivariate PLS for omics-type data. Adv Appl Bioinform Chem.

[29] Langfelder P, Horvath S (2008). WGCNA: an R package for weighted correlation network analysis. BMC Bioinform.

[30] Langfelder P, Zhang B, Horvath S (2008). Defining clusters from a hierarchical cluster tree: the dynamic tree cut package for R. Bioinformatics.

[31] Lee V, Camon E, Dimmer E, Barrell D, Apweiler R (2005). Who tangos with GOA? Use of gene ontology annotation (GOA) for biological interpretation of ‘-omics’ Data and for validation of automatic annotation tools. In Silico Biol.

[32] Arakawa K, Kono N, Yamada Y, Mori H, Tomita M (2002). KEGG-based pathway visualization tool for complex omics data. In Silico Biol.

[33] Bhargava P, Fitzgerald KC, Calabresi PA, Mowry EM (2017). Metabolic alterations in multiple sclerosis and the impact of vitamin D supplementation. JCI Insight.

[34] Su Y, Wang J, Shi M, Niu X, Yu X, Gao L, Zhang X, Lei C, Zhang W (2014). Metabolomic and network analysis of astaxanthin-producing *Haematococcus pluvialis* under various stress conditions. Bioresour Technol.

[35] Wang C, Huang D, Liang S (2018). Identification and metabolomic analysis of chemical elicitors for tacrolimus accumulation in *Streptomyces tsukubaensis*. Appl Microbiol Biotechnol.

[36] Novais FJ, Pires PRL, Alexandre PA, Dromms RA, Iglesias AH, Ferraz JBS, Styczynski MP, Fukumasu H (2019). Identification of a metabolomic signature associated with feed efficiency in beef cattle. BMC Genom.

[37] Reinforced Clostridial Medium. https://www.bd.com/europe/regulatory/Assets/IFU/Difco_BBL/218081.pdf. Accessed 25 Apr 2019.

[38] Wang B, Liu J, Liu H, Huang D, Wen J (2015). Comparative metabolic profiling reveals the key role of amino acids metabolism in the rapamycin overproduction by *Streptomyces hygroscopicus*. J Ind Microbiol Biotechnol.

[39] Xia M, Huang D, Li S, Wen J, Jia X, Chen Y (2013). Enhanced FK506 production in *Streptomyces tsukubaensis* by rational feeding strategies based on comparative metabolic profiling analysis. Biotechnol Bioeng.

[40] Pluskal T, Castillo S, Villar-Briones A, Oresic M (2010). MZmine 2: modular framework for processing, visualizing, and analyzing mass spectrometry-based molecular profile data. BMC Bioinform.

[41] Zhang B, Horvath S (2005). A general framework for weighted gene co-expression network analysis. Stat Appl Genet Mol.

[42] Pei G, Chen L, Zhang W (2017). Chapter nine-WGCNA application to proteomic and metabolomic data analysis.

[43] Martinez-Moya P, Niehaus K, Alcaino J, Baeza M, Cifuentes V (2015). Proteomic and metabolomic analysis of the carotenogenic yeast *Xanthophyllomyces dendrorhous* using different carbon sources. BMC Genom.

[44] Kruger NJ (1994). The Bradford method for protein quantitation. Methods Mol Biol.

[45] Shevchenko A, Jensen ON, Podtelejnikov AV, Sagliocco F, Wilm M, Vorm O, Mortensen P, Shevchenko A, Boucherie H, Mann M (1996). Linking genome and proteome by mass spectrometry: large-scale identification of yeast proteins from two dimensional gels. Proc Natl Acad Sci USA.

[46] Wang C, Chen J, Hu WJ, Liu JY, Zheng HL, Zhao F (2014). Comparative proteomics reveal the impact of OmcA/MtrC deletion on *Shewanella oneidensis* MR-1 in response to hexavalent chromium exposure. Appl Microbiol Biotechnol.

[47] Grupe H, Gottschalk G (1992). Physiological events in *Clostridium acetobutylicum* during the shift from acidogenesis to solventogenesis in continuous culture and presentation of a model for shift induction. Appl Environ Microb.

[48] Taherzadeh MJ, Gustafsson L, Niklasson C, Liden G (1999). Conversion of furfural in aerobic and anaerobic batch fermentation of glucose by *Saccharomyces cerevisiae*. J Biosci Bioeng.

[49] Lopez MJ, Nichols NN, Dien BS, Moreno J, Bothast RJ (2004). Isolation of microorganisms for biological detoxification of lignocellulosic hydrolysates. Appl Microbiol Biotechnol.

[50] Tishkov VI, Popov VO (2006). Protein engineering of formate dehydrogenase. Biomol Eng.

[51] Maddox IS, Steiner E, Hirsch S, Wessner S, Gutierrez NA, Gapes JR, Schuster KC (2000). The cause of “acid-crash” and “acidogenic fermentations” during the batch acetone-butanol-ethanol (ABE-) fermentation process. J Mol Microb Biotechnol.

[52] Dietmair S, Hodson MP, Quek LE, Timmins NE, Chrysanthopoulos P, Jacob SS, Gray P, Nielsen LK (2012). Metabolite profiling of CHO cells with different growth characteristics. Biotechnol Bioeng.

[53] Fujita Y, Freese E (1979). Purification and properties of fructose-1,6-bisphosphatase of *Bacillus subtilis*. J Biol Chem.

[54] Donahue JL, Bownas JL, Niehaus WG, Larson TJ (2000). Purification and characterization of glpX-encoded fructose 1, 6-bisphosphatase, a new enzyme of the glycerol 3-phosphate regulon of *Escherichia coli*. J Bacteriol.

[55] Gheshlaghi R, Scharer JM, Moo-Young M, Chou CP (2009). Metabolic pathways of *clostridia* for producing butanol. Biotechnol Adv.

[56] Crown SB, Indurthi DC, Ahn WS, Choi J, Papoutsakis ET, Antoniewicz MR (2011). Resolving the TCA cycle and pentose-phosphate pathway of *Clostridium acetobutylicum* ATCC 824: isotopomer analysis, in vitro activities and expression analysis. Biotechnol J.

[57] Amador-Noguez D, Brasg IA, Feng XJ, Roquet N, Rabinowitz JD (2011). Metabolome remodeling during the acidogenic-solventogenic transition in *Clostridium acetobutylicum*. Appl Environ Microbiol.

[58] Zhao J, Zhong C (2009). A review on research progress of transketolase. Neurosci Bull.

[59] Seo JS, Chong H, Park HS, Yoon KO, Jung C, Kim JJ, Hong JH, Kim H, Kim JH, Kil JI, Park CJ, Oh HM, Lee JS, Jin SJ, Um HW, Lee HJ, Oh SJ, Kim JY, Kang HL, Lee SY, Lee KJ, Kang HS (2005). The genome sequence of the ethanologenic bacterium *Zymomonas mobilis* ZM4. Nat Biotechnol.

[60] Conway T (1992). The Entner–Doudoroff pathway: history, physiology and molecular biology. FEMS Microbiol Rev.

[61] McGuire KA, Siggaard-Andersen M, Bangera MG, Olsen JG, Wettstein-Knowles P (2001). beta-Ketoacyl-[acyl carrier protein] synthase I of *Escherichia coli*: aspects of the condensation mechanism revealed by analyses of mutations in the active site pocket. Biochemistry.

[62] Choi KH, Heath RJ, Rock CO (2000). beta-Ketoacyl-acyl carrier protein synthase III (FabH) is a determining factor in branched-chain fatty acid biosynthesis. J Bacteriol.

[63] Yu XH, Prakash RR, Sweet M, Shanklin J (2014). Coexpressing *Escherichia coli* cyclopropane synthase with *Sterculia foetida* lysophosphatidic acid acyltransferase enhances cyclopropane fatty acid accumulation. Plant Physiol.

[64] Herrmann K, Entus R (2001). Shikimate pathway: aromatic amino acids and beyond.

[65] Schoner R, Herrmann KM (1976). 3-Deoxy-d-arabino-heptulosonate 7-phosphate synthase. Purification, properties, and kinetics of the tyrosine-sensitive isoenzyme from *Escherichia coli*. J Biol Chem.

[66] Junge W, Nelson N (2015). ATP synthase. Annu Rev Biochem.

[67] Gottwald M, Gottschalk G (1985). The internal pH of *Clostridium acetobutylicum* and its effect on the shift from acid to solvent formation. Arch Microbiol.

[68] Jones DT, Woods DR (1986). Acetone-butanol fermentation revisited. Microbiol Rev.

[69] Choi J, Choi S, Choi J, Cha MK, Kim IH, Shin W (2003). Crystal structure of Escherichia coli thiol peroxidase in the oxidized state: insights into intramolecular disulfide formation and substrate binding in atypical 2-Cys peroxiredoxins. J Biol Chem.

[70] Kawasaki S, Watamura Y, Ono M, Watanabe T, Takeda K, Niimura Y (2005). Adaptive responses to oxygen stress in obligatory anaerobes *Clostridium acetobutylicum* and *Clostridium aminovalericum*. Appl Environ Microbiol.

[71] Lipps G, Krauss G (1999). Adenylosuccinate synthase from *Saccharomyces cerevisiae*: homologous overexpression, purification and characterization of the recombinant protein. Biochem J.

[72] Carr DF, Whiteley G, Alfirevic A, Pirmohamed M (2009). Fol Ast: investigation of inter-individual variability of the one-carbon folate pathway: a bioinformatic and genetic review. Pharmacogenomics J.

[73] Alsaker KV, Papoutsakis ET (2005). Transcriptional program of early sporulation and stationary-phase events in *Clostridium acetobutylicum*. J Bacteriol.

[74] Long S, Jones DT, Woods DR (1984). Initiation of solvent production, clostridial stage and endospore formation in *Clostridium acetobutylicum* P262. Appl Microbiol Biotechnol.

[75] Durre P, Hollergschwandner C (2004). Initiation of endospore formation in *Clostridium acetobutylicum*. Anaerobe.

[76] Nolling J, Breton G, Omelchenko MV, Makarova KS, Zeng Q, Gibson R, Lee HM, Dubois J, Qiu D, Hitti J, Wolf YI, Tatusov RL, Sabathe F, Doucette-Stamm L, Soucaille P, Daly MJ, Bennett GN, Koonin EV, Smith DR (2001). Genome sequence and comparative analysis of the solvent-producing bacterium *Clostridium acetobutylicum*. J Bacteriol.

[77] Keweloh H, Weyrauch G, Rehm HJ (1990). Phenol-induced membrane changes in free and immobilized *Escherichia coli*. Appl Microbiol Biotechnol.

[78] Santos PM, Benndorf D, Sa-Correia I (2004). Insights into *Pseudomonas putida* KT2440 response to phenol-induced stress by quantitative proteomics. Proteomics.

